# The regulation of rhythmic locomotion by motor cortical and dopaminergic inputs in the mouse striatum

**DOI:** 10.1186/s13041-025-01232-8

**Published:** 2025-07-16

**Authors:** Hua Zhang, Yunxiao Su, Xujun Wu, Wen-Biao Gan

**Affiliations:** 1 School of Chemical Biology and Biotechnology, Peking University Shenzhen Graduate School, Shenzhen, 518055 China; 2 Lingang Laboratory, Shanghai, China; 3 Institute of Neurological and Psychiatric Disorders, Shenzhen Bay Laboratory, Shenzhen, 518132 China

**Keywords:** Striatum, Motor cortex, Dopaminergic receptors, Motor training, Locomotion, Imaging

## Abstract

The striatum is a critical component of the basal ganglia and plays a central role in regulating motor initiation and action selection. How cortical and subcortical inputs converging at the striatum regulate locomotion remains unclear. By examining gait changes in head-fixed mice running on a treadmill, we found that mice were capable of performing forward, but not backward, rhythmic locomotion using their forelimbs when the striatum and motor cortex were inactivated. The striatal activity is critical for adjusting initially disorganized gait to efficient rhythmic locomotion during forward running training, as well as for increasing the stride width during forward locomotion. The inputs from the motor cortex to striatum are important for the rhythmic locomotion, but not for changes of stride length and width during forward running training. In addition, D1 and D2 dopamine receptor activity in striatum are both important for efficient rhythmic locomotion, while exerting opposite effects on the stride width. Together, these results reveal multifactorial control of efficient and rhythmic gait by motor cortical and dopaminergic inputs converging at the striatum.

**Supplementary Information:**

The online version contains supplementary material available at 10.1186/s13041-025-01232-8.

## Introduction

The control of locomotion is a highly coordinated process that involves the integration of neural signals across multiple brain regions. The executive locomotor circuits that control the coordination of muscle activity are localized in the spinal cord [1–3], and the commands for movement initiation and gait selection come from different supraspinal structures, including cortical motor areas and the basal ganglia [4–8].

The striatum, a key component of the basal ganglia, plays a crucial role in the regulation of locomotion by integrating cortical, thalamic, and dopaminergic inputs to regulate the mesencephalic locomotor region (MLR), and central pattern generators (CPGs) in the spinal cord [9, 10]. As a key node in cortico-basal ganglia-thalamocortical circuits, the striatum contributes to movement initiation, action selection, and habit formation [11–14]. Impaired striatal output disrupts MLR and CPG function, leading to locomotor deficits as seen in Parkinson’s disease (PD) and Huntington’s disease (HD) [9, 15–17].

Glutamatergic inputs from the motor cortex project topographically to medium spiny neurons (MSNs) in the striatum [18, 19], enabling the striatum to integrate commands from motor cortex and other brain regions to regulate locomotor behaviors [20, 21]. In addition to locomotion, the motor corticostriatal pathway also plays important roles in motor learning and habit formation [19, 22–25]. Dysfunction in this pathway results in reduced cortical drive to the striatum and may impair locomotor control as seen in freezing of gait (FOG) of PD and the mild premanifest bradykinesia in HD [26–28].

In addition to motor cortical inputs to the striatum, dopaminergic inputs in the striatum, via D1 and D2 receptor-expressing MSNs, is essential for locomotor control. Direct pathway striatal projection neurons (dSPNs) primarily express D1-dopamine receptors, whereas indirect pathway projection neurons (iSPNs) primarily express D2-dopamine receptors [29, 30]. The dSPNs and iSPNs have opposite effects on activity in basal ganglia output structures, leading to different influences on action initiation and execution [31–33]. Similar to the corticostriatal pathway, D1-MSNs and D2-MSNs also play a crucial part in locomotor learning and habit formation [20, 34–36]. Selective stimulation of D1-MSNs increases locomotor activity, while their inhibition leads to motor suppression [32], and dysfunction in the direct pathway is implicated in hypokinetic disorders such as PD [37–39]. D2-MSNs can inhibit involuntary movements by counterbalancing the excitatory effects of the direct pathway. Loss of D2-MSNs appears to be involved in the chorea seen in early symptomatic HD [28, 40]. Although motor cortical inputs and dopaminergic inputs converging at the striatum are important for locomotor behaviors, how these inputs regulate various aspects of locomotion remains unclear.

In this study, we investigated how motor cortical and dopaminergic inputs to the striatum affect forelimb locomotion of head-fixed mice running on a treadmill. We found that mice without striatum activity were capable of performing forward (FW), but not backward (BW), rhythmic locomotion. Motor cortical and dopaminergic inputs to the striatum are critical for achieving efficient, rhythmic and stable gait during training.

## Results

### Mice lacking striatal and motor cortical activity can still generate FW rhythmic forelimb locomotion

**Fig. 1:**
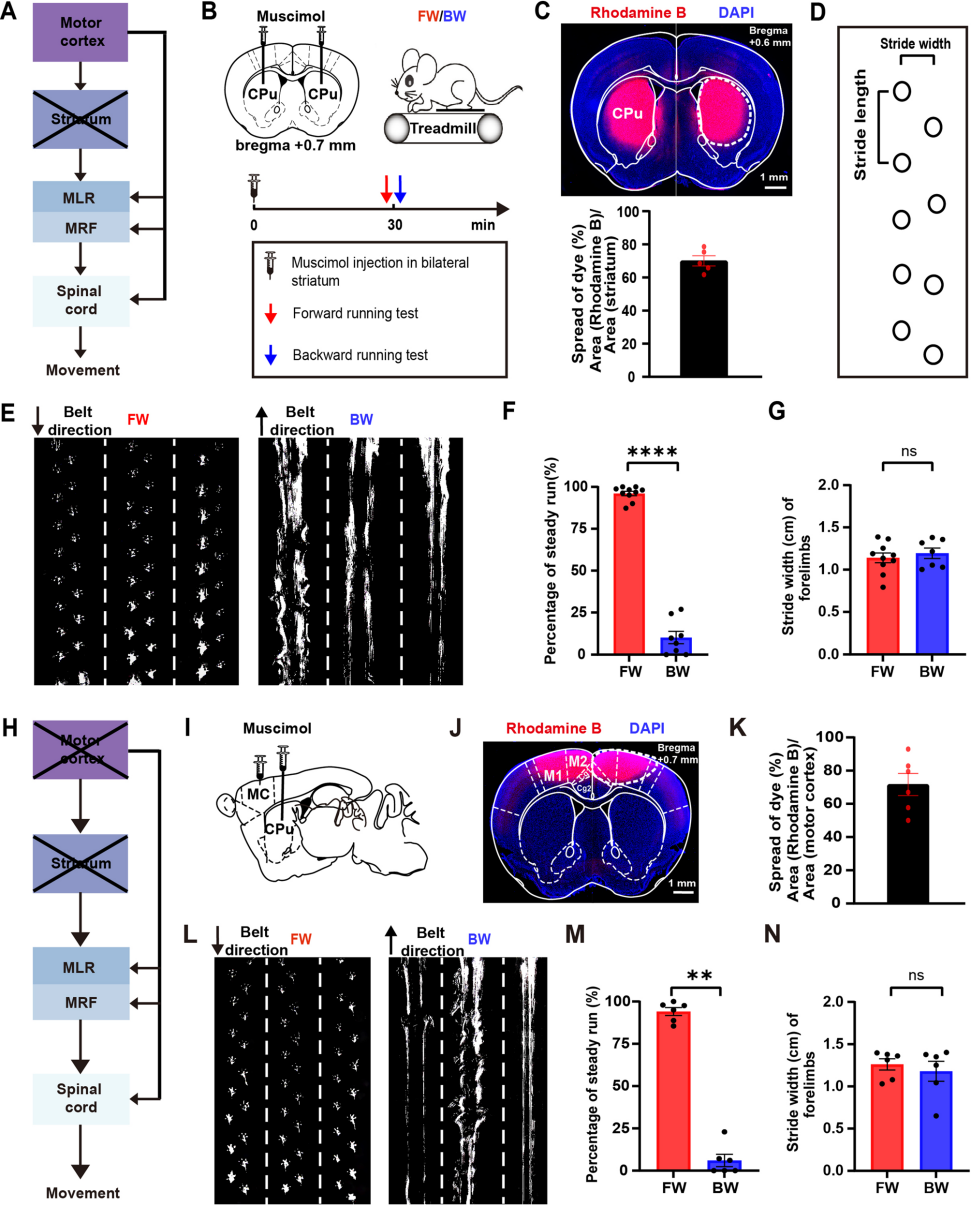
Mice lacking striatum and motor cortex activity can still produce FW rhythmic forelimb locomotion. (**A**) Schematic of motor circuits involved in locomotion regulation (slanted black lines represented inactivation of the striatum). (**B**) Top: Schematic of injecting muscimol into the bilateral striatum (CPu) (left). Schematic of FW and BW locomotion of forelimbs in mice on the treadmill (right). Bottom: Experimental design to test the effect of striatum inactivation on FW and BW locomotion. (**C**) Top: A representative brain section showing rhodamine B (to mimic muscimol) diffusion in the striatum 30 min after rhodamine B injection (dotted line represents the diffusion region of rhodamine B). Scale bar, 1 mm. Bottom: The ratio of rhodamine B diffusion area relative to the striatum area in the representative section showing the largest dye spread (*n* = 6 mice). (**D**) Schematic of analyzing the stride width and stride length of left forelimbs in mice (black circle represents the footprints of forelimbs in mice). (**E**) Examples of FW (left) and BW (right) locomotion performance 30 min after inactivating neuronal activity in the striatum (dotted lines separate gait in 3 individual trials). (**F**) The percentage of steady run during FW locomotion was significantly higher than BW locomotion 30 min after inactivation of the striatum (FW = 10 mice; BW = 8 mice). (**G**) The stride width of forelimbs had no significant difference between FW and BW locomotion 30 min after inactivating the striatum (FW = 10 mice; BW = 7 mice). (**H**) Schematic of motor circuits involved in locomotion regulation (slanted black lines represented inactivation of both the striatum and motor cortex). (**I**) Schematic of injecting muscimol to inactivate both the striatum and motor cortex (MC). (**J**) A representative brain section showing rhodamine B (to mimic muscimol) diffusion in the motor cortex 30 min after rhodamine B injection (dotted line represents the diffusion region of rhodamine B). Scale bar, 1 mm. (**K**) The ratio of rhodamine B diffusion area relative to the motor cortex area in the representative section showing the largest dye spread (*n* = 5 mice). (**L**) Examples of FW (left) and BW (right) locomotion performance 30 min after inactivating neuronal activity of both the striatum and motor cortex. (**M**) The percentage of steady run during FW locomotion was significantly higher than BW locomotion 30 min after inactivation of both the striatum and motor cortex (FW = 6 mice; BW = 6 mice). (**N**) The stride width of forelimbs had no significant difference between FW and BW locomotion 30 min after inactivating the striatum and motor cortex (FW = 6 mice; BW = 6 mice). All data are presented as mean ± S.E.M. **P < 0.01, ****P < 0.0001, ns = no significant difference (*P* > 0.05). (F, G, M, N): Mann-Whitney test

Many lines of evidence have shown that the striatum modulates locomotor activity through disinhibitory mechanisms involving the substantia nigra pars reticulata (SNr) and the globus pallidus internus (GPi), which in turn regulate the activity of the MLR and CPGs [1, 41–43]. On the other hand, electric stimulation of the brain stem of decerebrate cats is capable of triggering basic stepping movements, suggesting that the MLR and CPGs could generate rhythmic locomotion [44, 45]. To better understand the function of the striatum and its downstream motor circuits in regulating locomotion, we first examined the gait of head-fixed mice running forward (FW) or backward (BW) on a treadmill with forelimbs after injecting 250 nL of muscimol (1 µg/µL) into the center of striatum (Fig. 1A and B). The use of rhodamine B dye to mimic muscimol diffusion suggested that more than 60% of the striatum area was affected by muscimol 30 min after the injection (Fig. 1C and Supplementary Fig. 1). Importantly, when Ca^2+^ activity of GCaMP8-expressing striatal neurons was imaged with fiber photometry 30 min after muscimol injection (see the method), Ca^2+^ activity of striatal neurons was significantly reduced by ∼ 96.08% (Supplementary Fig. 2, ΔF/F_0_ (0 min): 214.50 ± 13.32; ΔF/F_0_ (30 min): 8.41 ± 2.81, 9 trials, *n* = 3 mice, *P* < 0.01).

When the gait of FW or BW running on the treadmill was examined, we found that mice with bilaterally inactivated striatum could perform FW, but not BW, rhythmic locomotion (Fig. 1D and F). FW running primarily consisted of steady run, characterized by periodic footsteps with clear paw prints on the treadmill, whereas BW running mostly involved drags with no foot lifting (Fig. 1E). The percentage of steady run in FW locomotion was significantly higher than in BW locomotion (Fig. 1F, FW: 96.08 ± 1.34%, *n* = 10; BW: 10.24 ± 3.69%, *n* = 8, *P* < 0.0001). The stride width between two forelimbs had no significant difference between FW and BW locomotion (Fig. 1G; FW: 1.14 ± 0.06, *n* = 10; BW: 1.19 ± 0.06, *n* = 7, *P* = 0.81).

To determine whether in the absence of striatal activity, the motor cortex might be involved in FW rhythmic locomotion of the forelimbs, we injected muscimol into both the striatum and motor cortex (including the primary and secondary motor cortex) and examined FW or BW locomotion of forelimbs on the treadmill (Fig. 1H and I). We found that rhodamine B (mimicking muscimol diffusion) was spread across more than 70% of the motor cortex area 30 min after the injection (Fig. 1J and K, Supplementary Fig. 1). Furthermore, Ca^2+^ imaging showed that somatic Ca^2+^ activity of pyramidal neurons was substantially decreased 30 min after muscimol injection in the motor cortex (Supplementary Fig. 3, ΔF/F_0_ (0 min): 22.36 ± 2.37; ΔF/F_0_ (30 min): 1.37 ± 0.08, 164 cells, *n* = 4 mice, *P* < 0.0001). Importantly, with muscimol inhibition of both striatum and motor cortex activity, mice could still perform the FW, but not BW, rhythmic locomotion. The percentage of steady run in FW locomotion was significantly higher than BW locomotion (Fig. 1L and M, FW: 94.14 ± 2.34%, *n* = 6; BW: 6.03 ± 3.63%, *n* = 6, *P* < 0.01). The stride width between two forelimbs had no significant difference between FW and BW locomotion (Fig. 1L and N, FW: 1.26 ± 0.07, *n* = 6; BW: 1.18 ± 0.12, *n* = 6, *P* = 0.82).

Taken together, these results indicate that the striatum and motor cortex are unnecessary for mice to perform FW rhythmic locomotion of the forelimbs on the treadmill but are important for BW rhythmic locomotion. They also suggest that MLR and spinal cord CPG regions downstream of the striatum and motor cortex are capable of generating FW rhythmic locomotion of forelimbs on the treadmill.

### Striatal activity is important for efficient rhythmic locomotion following FW running training

**Fig. 2:**
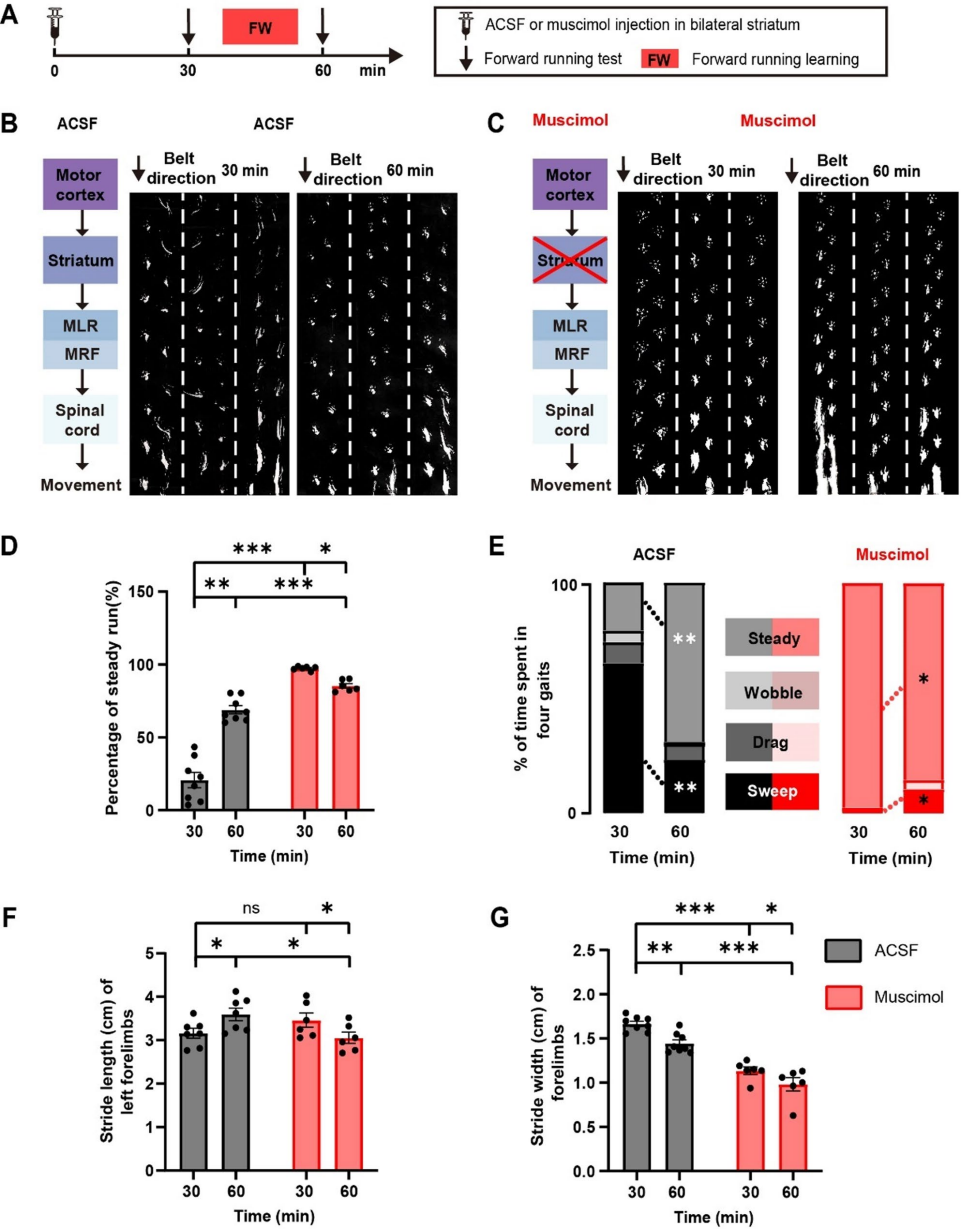
Striatal activity is important for efficient rhythmic locomotion following FW running training. (**A**) Experimental design to test the effect of neuronal activity in the striatum on FW locomotion before and after training. (**B**) Examples of FW locomotion performance before (left) and after (right) 30-minute FW running training in control mice with ACSF injection in the bilateral striatum (dotted lines separate gait in 3 individual trials). (**C**) Examples of FW locomotion performance before (left) and 30 min after (right) FW running training in striatal inactivation mice with muscimol injection in the bilateral striatum. (**D**) The percentage of steady run was low initially but increased significantly in the control mice (*n* = 8 mice) after 30-minute FW training. The percentage of steady run was high initially but reduced significantly in mice with muscimol injection (*n* = 6 mice) after 30-minute FW training. (**E**) Changes of forelimb gait before and after 30-minute FW training with (left) or without (right) striatal activity. Mice with striatal activity exhibit seemingly chaotic gait at the beginning of training. 30 min after FW training, mice show an increase in steady run and a decrease in sweep training (ACSF = 8 mice; Muscimol = 6 mice). (**F**) The stride length of forelimbs before and after 30-minute FW training. The stride length significantly increased after 30-minute FW training in the control mice (*n* = 7 mice), while inactivating the striatum caused a significant reduction in the stride length during FW training (*n* = 6 mice). (**G**) The stride width of forelimbs before and after 30-minutes FW training. The stride width in FW locomotion was significantly larger in control mice (*n* = 8 mice) than that in mice subjected to inactivating the striatum (*n* = 6 mice). All data are presented as mean ± S.E.M. *P < 0.05, **P < 0.01, ***P < 0.001, ns = no significant difference (*P* > 0.05). (D-G): Wilcoxon test and Mann-Whitney test. See methods for statistical details

While the findings above show that motor circuits downstream of the striatum can generate basic rhythmic FW locomotion, striatal activity likely fine-tunes gait parameters such as rhythmicity, stride length and width. To investigate this, we compared forelimb locomotion on a treadmill with or without inactivating the striatum 30 min after muscimol or ACSF injection (Fig. 2A).

Notably, while mice that received muscimol injection into the striatum showed rhythmic locomotion (Fig. 1E-F), control mice that received ACSF injection exhibited mostly disorganized gait and a significantly lower percentage of steady run (Fig. 2B-D, ACSF (30 min): 20.73 ± 5.23%, *n* = 8; Muscimol (30 min): 97.42 ± 0.54%, *n* = 6, *P* < 0.001). Detailed examination of gait showed that mice without striatal activity exhibited mostly steady run throughout running trials, while mice with striatum activity exhibited mostly sweep, drag and wobble, in addition to a small percentage of steady run (Fig. 2E, ACSF, Muscimol at 30 min). These results suggest that striatal activity induces variability on top of basic rhythmic FW locomotion generated by the downstream motor circuits.

To determine whether and how the striatum is involved in gait changes during FW motor training, mice were trained to run FW on the treadmill with forelimbs over a period of 30 min with or without striatum inactivation (Fig. 2A). We found that mice with striatal activity showed improvement of steady run and reduced sweep pattern 30 min after treadmill training (Fig. 2D and E, Steady run (ACSF): 20.73 ± 5.23% (30 min); 68.99 ± 2.85% (60 min), *n* = 8, *P* < 0.01; Sweep (ACSF): 65.21 ± 5.66% (30 min); 23.24 ± 2.86% (60 min), *n* = 8, *P* < 0.01). In addition, the stride length showed a significant increase over 30-minute FW running training (Fig. 2F, ACSF: 3.16 ± 0.11 (30 min); 3.60 ± 0.14 (60 min), *n* = 7, *P* < 0.05). In contrast, mice with inactivated striatum showed a reduction in the percentage of steady run and increase in the sweep pattern (Fig. 2D and E, Steady run (Muscimol): 97.42 ± 0.54% (30 min); 85.40 ± 1.60% (60 min), *n* = 6, *P* < 0.05; Sweep (Muscimol): 1.85 ± 0.48% (30 min); 10.61 ± 1.88% (60 min), *n* = 6, *P* < 0.05). Inactivation of the striatum also caused a significant reduction in the stride length over 30-minute FW running training (Fig. 2F, Muscimol: 3.46 ± 0.16 (30 min); 3.06 ± 0.13 (60 min), *n* = 6, *P* < 0.05). Taken together, these results show that neuronal activity of the striatum is important for changes from initially disorganized gait to more organized ones, as well as for the increase of stride length, thereby achieving efficient rhythmic locomotion over FW running training.

Furthermore, 30 min after ACSF or muscimol injection, the stride width of forelimbs was significantly larger in mice with striatal activity as compared to mice with striatum inactivation during FW locomotion (Fig. 2G, ACSF (30 min): 1.66 ± 0.03, *n* = 8; Muscimol (30 min): 1.13 ± 0.04, *n* = 6, *P* < 0.001). Over 30-minute FW running training, a significant reduction in the stride width was observed in mice with or without striatal activity (Fig. 2G, ACSF (30 min): 1.66 ± 0.03; ACSF (60 min): 1.44 ± 0.04, *n* = 8, *P* < 0.01; Muscimol (30 min): 1.13 ± 0.04; Muscimol (60 min): 0.98 ± 0.07, *n* = 6, *P* < 0.05). Nevertheless, the stride width in mice with striatum activity was still significantly larger than that of mice without striatum activity after 30-minute FW training (Fig. 2G, *P* < 0.001).

Taken together, the findings above suggest that although striatal activity is dispensable for mice to perform FW rhythmic locomotion, it is important for introducing variabilities to rhythmic locomotion generated by MLR and spinal cord, allowing changes from initially disorganized and less efficient gait at the beginning of the FW running training to a more rhythmic and efficient one after the training. They also suggest that striatal activity is important for increasing the stride width during FW running, presumably facilitating stable locomotion on the treadmill.

### Motor cortical inputs to striatum are important for rhythmic locomotion, but not for changes in stride width and length, following FW treadmill training

**Fig. 3:**
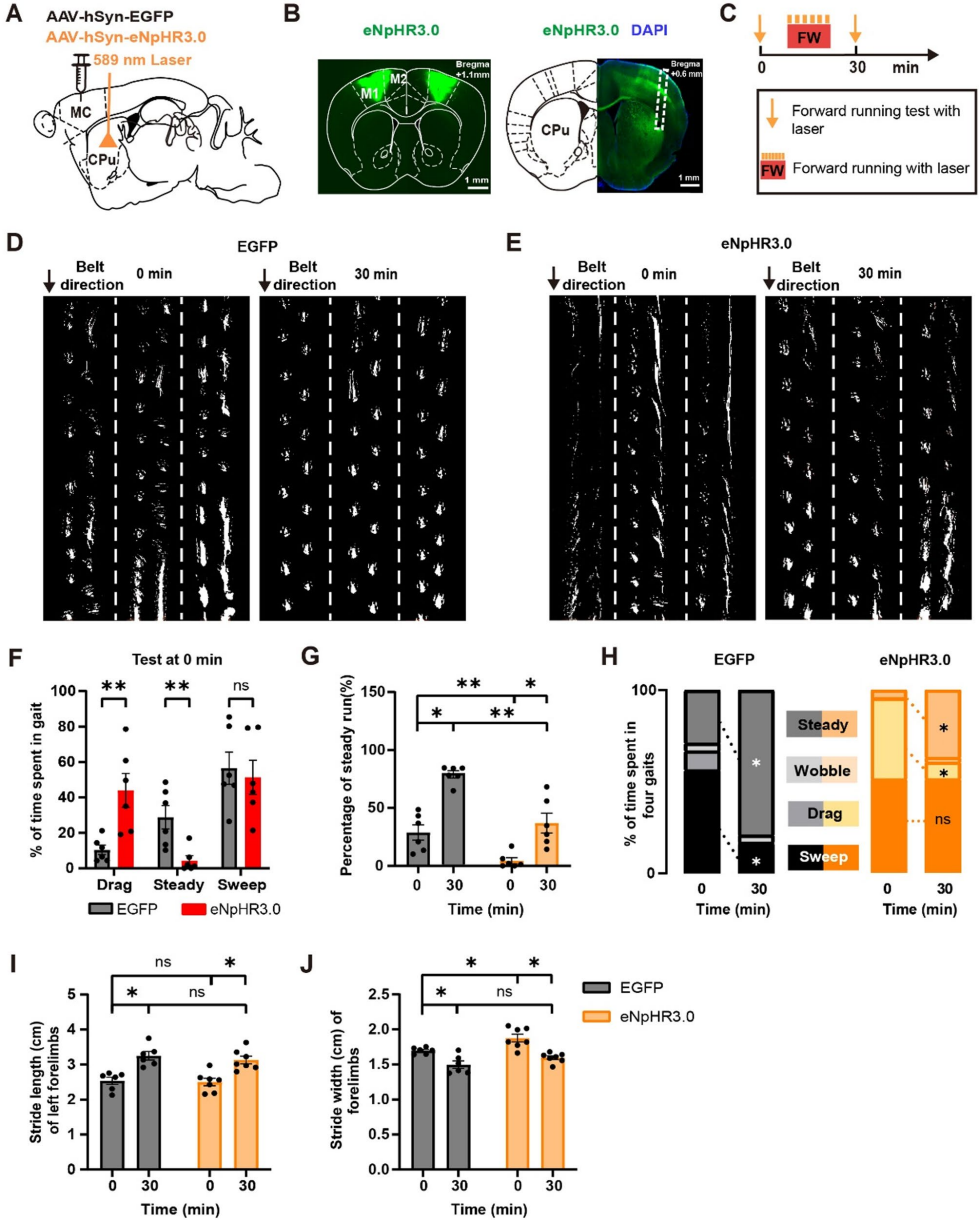
Motor cortical inputs to striatum are important for rhythmic locomotion, but not for stride width and length changes, following FW treadmill training. (**A**) Schematic of injecting halorhodopsin (eNpHR3.0) or control (EGFP) virus in the bilateral motor cortex to manipulate the activity of neuronal projections from the motor cortex to DLS. (**B**) A representative brain section of halorhodopsin virus expression in the bilateral motor cortex (left). Representative example of implanted optical fiber in bilateral DLS to inhibit the projections from motor cortex to DLS (right). (**C**) Experimental design. (**D-E**) Examples of FW locomotion performance before (left) and after (right) 30-minute FW running training in mice with (**E**) or without (**D**) inactivating motor cortical inputs to the DLS (dotted lines separate gait in 3 individual trials). (**F**) Inhibiting the inputs from the motor cortex to DLS significantly increased the drag percentage and decreased steady run percentage, but had no significant effect on sweep percentage during FW locomotion in mice before FW training (EGFP = 6 mice; eNpHR3.0 = 6 mice). (**G**) Inhibiting the inputs from the motor cortex to DLS significantly reduced the percentage of steady run as compared to the control after 30-minute FW training (EGFP = 6 mice; eNpHR3.0 = 6 mice). (**H**) Changes of forelimb gait before and after 30-minute FW training with (right) or without (left) inactivation of the motor corticostriatal pathway. Note the reduction of drag, not sweep, occurs after 30-minute FW training when the motor corticostriatal pathway was inhibited (EGFP = 6 mice; eNpHR3.0 = 6 mice). (**I**) Inhibiting the inputs from the motor cortex to DLS didn’t affect the stride length in FW locomotion before training and the increase in stride length following 30-minute FW training (EGFP = 6 mice; eNpHR3.0 = 7 mice). (**J**) Inhibiting the inputs from the motor cortex to DLS significantly increased the stride width before FW training but didn’t affect the decrease of stride width following FW training (EGFP = 6 mice; eNpHR3.0 = 7 mice). All data are presented as mean ± S.E.M. *P < 0.05, **P < 0.01, ns = no significant difference (*P* > 0.05). (D-H): Wilcoxon test and Mann-Whitney test. See methods for statistical details

Motor cortex is believed to be critical for the acquisition and execution of motor skills [46–49]. In addition to the direct projections to MLR and spinal cord, previous studies have shown that motor cortex sends dense projections to the striatum [18, 19, 50]. This motor corticostriatal pathway plays important roles in locomotion, motor learning and habit formation [22, 24]. Consistently, when the output neurons of the motor cortex were labeled with GFP, extensive projections from the motor cortex were observed in the dorsal lateral striatum (DLS) (Fig. 3A and B).

To investigate the function of the motor corticostriatal pathway in FW locomotion, we used the optogenetic approach to inhibit neuronal projections from the motor cortex to DLS (Fig. 3A and B). In this experiment, halorhodopsin virus (AAV-hsyn-eNpHR3.0) or control virus (AAV-hsyn-EGFP) was expressed bilaterally in the primary motor cortex (see the method). Two weeks after virus expression, we implanted optical fibers bilaterally in DLS and used 589 nm yellow light to inactivate axonal projections from the motor cortex to DLS after one-week recovery (Fig. 3A and B).

We found that the optogenetic inhibition of the motor cortex to the DLS pathway significantly affected FW locomotion, as evidenced by an increase in drag and a decrease in steady run as compared to control mice (Fig. 3C-F, Drag (EGFP): 10.40 ± 2.73%, *n* = 6; Drag (eNpHR3.0): 44.01 ± 9.57%, *n* = 6, *P* < 0.01; Steady (EGFP): 28.83 ± 6.61%, *n* = 6; Steady (eNpHR3.0): 4.40 ± 2.69%, *n* = 6, *P* < 0.01). There was no significant difference in the percentage of sweep between mice with and without optogenetic inhibition (Fig. 3F, Sweep (EGFP): 56.51 ± 9.11%, *n* = 6; Sweep (eNpHR3.0): 51.38 ± 9.59%, *n* = 6, *P* = 0.48). These results indicate that this motor corticostriatal pathway is involved in reducing drag and promoting steady run during FW locomotion.

Interestingly, after 30 min of FW run training, the percentage of drag significantly decreased and steady run significantly increased in mice with inactivation of the motor corticostriatal pathway (Fig. 3G and H, Drag (eNpHR3.0): 44.01 ± 9.57% (0 min); 9.14 ± 4.56% (30 min), *n* = 6, *P* < 0.05; Steady (eNpHR3.0): 4.40 ± 2.69% (0 min); 36.90 ± 8.57% (30 min), *n* = 6, *P* < 0.05). Nevertheless, the percentage of steady run was still significantly lower as compared to the control mice 30 min after FW training (Fig. 3G, Steady (EGFP): 28.83 ± 6.61% (0 min); 78.95 ± 3.11% (30 min), *n* = 6, *P* < 0.05; EGFP (30 min) vs. eNpHR3.0 (30 min), *P* < 0.01). Furthermore, there was a significant decrease in the percentage of sweep in control mice, but not in mice with optogenetic inhibition (Fig. 3H, Sweep (EGFP): 56.51 ± 9.11% (0 min); 16.49 ± 1.60% (30 min), *n* = 6, *P* < 0.05; Sweep (eNpHR3.0): 51.38 ± 9.59% (0 min); 51.46 ± 10.56% (30 min), *n* = 6, *P* > 0.99). Thus, without the motor cortical inputs to the DLS, mice could show partial, but not full, improvement in gait after 30-minute FW run training.

In addition, we found that inactivating the motor corticostriatal pathway didn’t affect the stride length (Fig. 3I, EGFP (0 min): 2.54 ± 0.10, *n* = 6; eNpHR3.0 (0 min): 2.50 ± 0.11, *n* = 7, *p* = 0.53), but significantly increased the stride width before FW training (Fig. 3J, EGFP (0 min): 1.70 ± 0.02, *n* = 6; eNpHR3.0 (0 min): 1.88 ± 0.05, *n* = 7, *P* < 0.05). Following 30-minute FW training, inhibiting the inputs from the motor cortex to DLS didn’t affect the increase of the stride length (Fig. 3I, EGFP: 2.54 ± 0.10 (0 min); 3.25 ± 0.12 (30 min), *n* = 6, *P* < 0.05; eNpHR3.0: 2.50 ± 0.11 (0 min); 3.13 ± 0.11 (30 min), *n* = 7, *P* < 0.05) nor the decrease of the stride width (Fig. 3J, EGFP: 1.70 ± 0.02 (0 min); 1.50 ± 0.05 (30 min), *n* = 6, *P* < 0.05; eNpHR3.0: 1.88 ± 0.05 (0 min); 1.60 ± 0.03 (30 min), *n* = 7, *P* < 0.05).

Together, these findings show that the motor corticostriatal pathway is important for increasing steady run by reducing drag and sweep during FW run training. In addition, while the motor cortical inputs to the striatum are involved in reducing the stride width during locomotion, they are not important for changes of the stride width and length over 30-minute FW run training.

### D1 and D2 dopamine receptor activity in the striatum are important for efficient and rhythmic locomotion

Many lines of evidence suggest that the striatal D1 and D2 MSNs play critical and opposing roles in regulating locomotor behavior [31, 32, 51]. The balance between D1 receptors (direct pathway) and D2 receptors (indirect pathway) activity is crucial for normal locomotion [14, 52]. While the activation of the indirect pathway results in freezing and bradykinesia, activating the direct pathway significantly improve these symptoms [32].

**Fig. 4:**
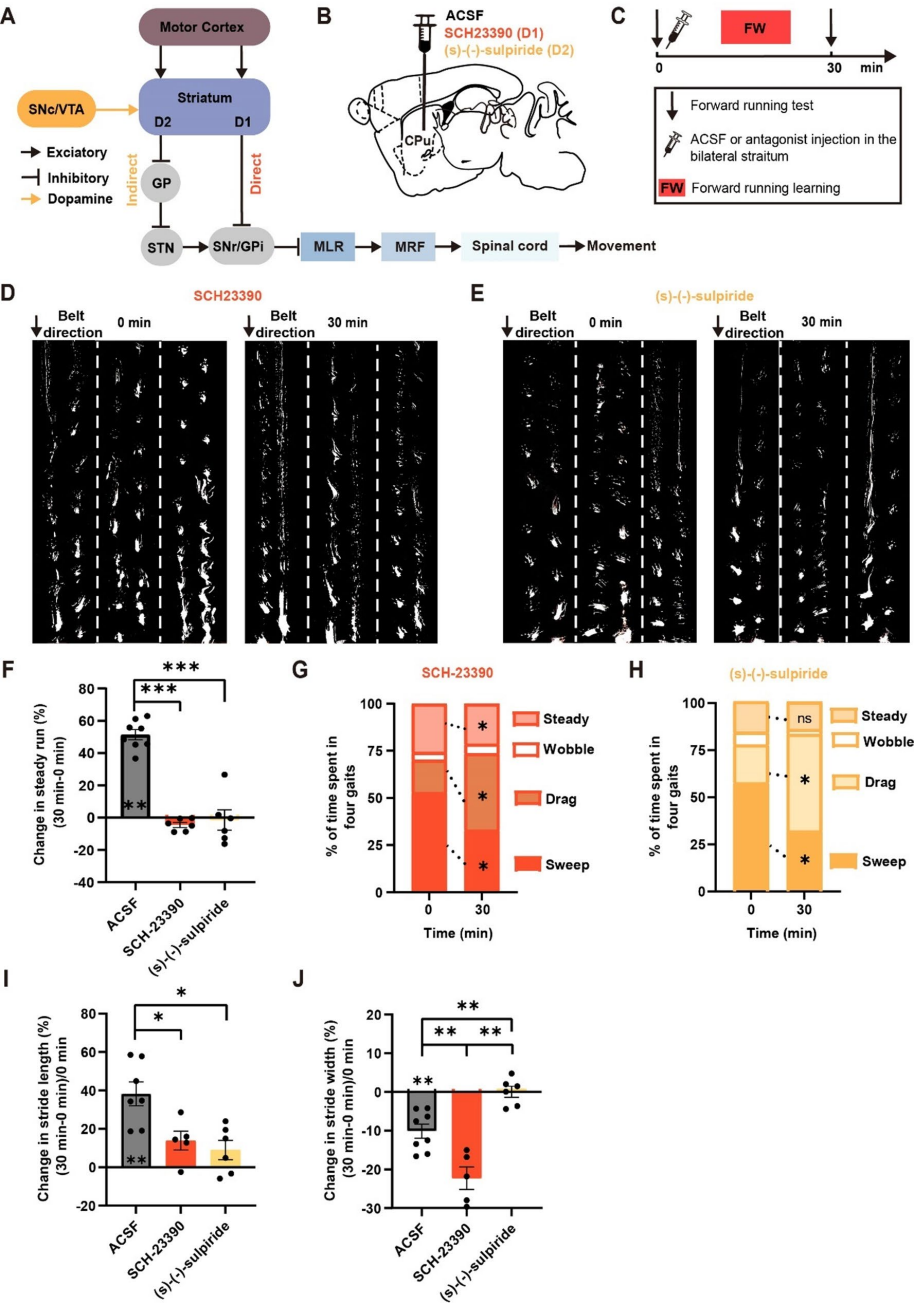
D1 and D2 dopamine receptor activity in the striatum are important for efficient and rhythmic locomotion. (**A**) Schematic of direct pathway (D1-dopamine receptor) and indirect pathway (D2-dopamine receptor) involved in motor control. (**B**) Schematic of injecting ACSF, D1 receptor antagonist SCH-23390 or D2 receptor antagonist (s)-(-)-sulpiride in the striatum bilaterally. (**C**) Experimental design. (**D-E**) Examples of FW locomotion performance before (left) and after (right) 30-minute FW running training after inhibiting dopamine receptor D1 (**D**) and D2 (**E**) receptor activity of the bilateral striatum (dotted lines separate gait in 3 individual trials). (**F**) Inhibiting D1 receptor or D2 receptor activity in the bilateral striatum prevented the increase in steady run after 30-minute FW training (ACSF = 8 mice; SCH-23390 = 6 mice; (s)-(-)-sulpiride = 6 mice). (**G-H**) Changes of forelimb gait before and after 30-minute FW training after inhibiting D1 (**G**) or D2 (**H**) receptor activity in the bilateral striatum. Inhibiting D1 or D2 receptor activity in the bilateral striatum significantly increased the drag pattern during FW training (SCH-23390 = 6 mice; (s)-(-)-sulpiride = 6 mice). (**I**) Inhibiting D1 or D2 receptor activity in the bilateral striatum prevented the increase of stride length following FW training (ACSF = 7 mice; SCH-23390 = 5 mice; (s)-(-)-sulpiride = 6 mice). (**J**) Inhibiting D1 receptor activity, but not D2 receptor activity, in the bilateral striatum caused a significant reduction in the stride width after 30-minute FW training (ACSF = 8 mice; SCH-23390 = 5 mice; (s)-(-)-sulpiride = 6 mice). All data are presented as mean ± S.E.M. *P < 0.05, **P < 0.01, ***P < 0.001, ns = no significant difference (*P* > 0.05). (F-J): Wilcoxon test and Mann-Whitney test. See methods for statistical details

Our findings above indicate that striatal activity is important for the adjustment from disorganized locomotion to efficient rhythmic one during motor training. In addition, the motor cortical inputs to the DLS are important for the adjustment to rhythmic locomotion but not for changes in stride length and width during motor training. To investigate whether the aforementioned FW locomotion improvement after training depends on dopaminergic inputs, we used D1 receptor antagonist SCH-23390 or D2 receptor antagonist (s)-(-)-sulpiride to inhibit the receptor activity in the striatum bilaterally during 30 min FW running training (Fig. 4A and C).

We found that 30 min after FW training, the percentage of steady run significantly increased in control mice but not in mice with D1 or D2 receptor activity inhibited (Fig. 4D-F, ACSF: 51.40 ± 3.16%, *n* = 8; SCH-23390: -4.47 ± 1.53%, *n* = 6; (s)-(-)-sulpiride: -1.49 ± 6.30%, *n* = 6, ACSF vs. SCH-23390, *P* < 0.001; ACSF vs. (s)-(-)-sulpiride, *P* < 0.001). Detailed analysis showed that while control mice exhibited an increase in steady run and a decrease in sweep gait after 30 min FW training (Fig. 2E, ACSF), mice with inhibited D1 or D2 receptor activity showed an increase in drag and a decrease in sweep (Fig. 4G and H, SCH-233900 (Drag): 17.19 ± 8.80% (0 min); 40.59 ± 9.81% (30 min), *n* = 6, *P* < 0.05; SCH-233900 (Sweep): 53.01 ± 5.80% (0 min); 33.10 ± 6.24% (30 min), *n* = 6, *P* < 0.05; (s)-(-)-sulpiride (Drag), 20.21 ± 4.83% (0 min); 51.29 ± 8.74% (30 min), *n* = 6, *P* < 0.05; (s)-(-)-sulpiride (Sweep): 57.84 ± 5.22% (0 min); 32.37 ± 5.18% (30 min), *n* = 6, *P* < 0.05). The percentage of sweep or drag had no significant difference after 30-minute FW training between mice with D1 and D2 receptor inactivation (Sweep: *P* = 0.94; Drag: *P* = 0.48). Furthermore, we found that inhibiting D1 or D2 receptor activity significantly reduced the stride length increase after FW training (Fig. 4I, ACSF: 38.28 ± 6.21%, *n* = 7; SCH-23390: 13.88 ± 4.94%, *n* = 5; (s)-(-)-sulpiride: 9.02 ± 5.03%, *n* = 6; ACSF vs. SCH-23390, *P* < 0.05; ACSF vs. (s)-(-)-sulpiride, *P* < 0.05). Taken together, these findings show that during FW run training, both the direct and indirect pathway are important for increasing steady run by suppressing the action of drag, as well as increasing the stride length, thereby facilitating efficient and rhythmic locomotion.

As expected, 30 min after FW training, the control mice with ACSF injection in the striatum showed a significant reduction in the stride width (Fig. 4J, 0 min: 1.56 ± 0.06; 30 min: 1.40 ± 0.06, *n* = 8, *P* < 0.01). Interestingly, inhibiting D1 receptor activity caused a significantly larger reduction in the stride width after 30-minute FW training, but inhibiting D2 receptor activity didn’t decrease it (Fig. 4J, ACSF: -10.12 ± 6.21%, *n* = 8; SCH-23390: -22.26 ± 2.91%, *n* = 5; (s)-(-)-sulpiride: 0.05 ± 1.43%, *n* = 6; ACSF vs. SCH-23390, *P* < 0.01; ACSF vs. (s)-(-)-sulpiride, *P* < 0.01). Thus, while D1 and D2 receptor activity in the striatum are important for efficient rhythmic locomotion, they exert opposite effect on the stride width change following FW locomotion training: D1 receptor activity is critical for increasing the stride width while D2 receptor activity is involved in reducing the stride width.

## Discussion

The striatum integrates inputs from the motor cortex, substantia nigra pars compacta (SNc) and ventral tegmental area (VTA) to regulate locomotion. Using optogenetic and pharmacological approaches to inhibit motor cortical inputs or dopaminergic receptor activity in the striatum, our study provides novel insights into the multifactorial control of rhythmic FW locomotion and locomotor refinement during motor training. While basic rhythmic locomotion can be generated by subcortical structures below the striatum and motor cortex, the motor cortical and dopaminergic inputs in the striatum play critical roles in modulating basic rhythmic gait to achieve efficient, rhythmic and stable locomotion during motor training.

Our results show that mice could perform FW, but not BW, rhythmic locomotion when the striatum and motor cortex were inactivated. Previous studies have shown that decerebrate cats or cats with spinal transection can still produce swallowing or walking on the treadmill [53–55]. Furthermore, electric stimulation of the brain stem of decerebrate cats can produce rhythmic stepping movements [44, 45, 56]. Consistently, the occurrence of FW rhythmic forelimb movement following inactivation of either the striatum alone or both the striatum and motor cortex in this study indicates that basic FW rhythmic locomotor patterns are generated by subcortical structures such as MLR in the brainstem and CPGs in the spinal cord [1, 2, 57, 58]. Although both FW and BW running with forelimbs on the treadmill are new motor experiences, FW locomotion is the most common and innate form of movement in both bipedal and quadrupedal animals, while BW locomotion is less familiar movement than FW locomotion. It has been shown that motor cortex is less engaged in the more proficient movement [59]. Therefore, it is possible that mice are less dependent on motor and striatum for rhythmic FW than BW locomotion because of their experience with FW movement throughout development.

The striatum is well known for its role in action selection and movement vigor control [11, 13, 60]. In our study, the striatal activity is critical for generating variability in gaits and increasing the stride width and length during locomotion training. These findings support a model in which the striatum acts as a “tuner” of locomotion, converting variable, exploratory movements into precise, rhythmic patterns through training. The observed narrow stride width and short stride length in the absence of striatal activity suggest that while motor circuits such as MLR and CPG downstream of striatum are sufficient for producing rhythmic locomotion, they lack the capacity to generate efficient and stable gait during motor locomotion.

Our studies show that inhibiting the motor cortical projections to the DLS affects the improvement of rhythmic locomotion as evidenced by the reduced percentage of steady run following FW training. Interestingly, inhibiting this motor corticostriatal pathway does not significantly affect the increase of stride length and the decrease of stride width during FW motor training, suggesting that this pathway is not critical for change of stride length and width in FW treadmill training. It is important to note that previous studies have shown that increasing inhibitory neuronal activity in the motor cortex (not specifically the activity in motor corticostriatal pathway) reduces the increase of steady run and causes changes in the stride length and width during FW motor training [47, 49]. In addition to the motor corticostriatal pathway, the motor cortex also sends direct projections to subcortical structures such as MLR, brainstem reticulospinal neurons, and spinal cord to regulate locomotion [61–65]. These direct projections are important for rapid adjustments to locomotion, such as obstacle avoidance or changes in gait, which require immediate integration of sensory feedback [61, 66, 67]. Therefore, it is possible that the direct motor cortical inputs to MLR and spinal cord are involved in the stride width decrease and stride length increase following FW training. Future studies are needed to specifically inactivate these direct pathways to determine whether and how these connections regulate various parameters of rhythmic locomotion.

It is generally believed that D1-MSNs (direct pathway) and D2-MSNs (indirect pathway) in the striatum play opposite roles in locomotor regulation: activating D1-MSNs promotes movement, whereas activating D2-MSNs suppresses unwanted movements [32, 68, 69]. Recent studies also suggest that inhibiting indirect pathway reduces locomotor activity and disturbs action termination and selection [13, 70]. Our findings show that both D1 and D2 dopaminergic inputs play a critical role in rhythmic locomotion by increasing steady run and reducing drag (unwanted movements). In addition, D1 and D2 dopaminergic inputs are also important for stride length increase following FW training. We further show that inhibiting D1 receptor activity decreases the stride width, while inhibiting D2 receptor activity increases it. These findings are consistent with the view that D1-MSNs and D2-MSNs in the striatum are not simple acting in opposite manner during locomotion, but are coordinated in action selection and locomotion refinement [14, 52].

Our findings demonstrate that motor cortical and dopaminergic inputs to the striatum critically regulate locomotion parameters, including the percentage of steady run, sweep and drag, as well as stride length and stride width. In mice with intact striatal function, initial sweep and drag behaviors likely reflect striatal-mediated trial-and-error adaptation to treadmill running. With training, this transitions to more efficient and stable rhythmic locomotion characterized by increased steady run and longer stride lengths. Notably, an optimal stride width is essential for maintaining locomotor stability [71]. Both excessively wide and narrow step widths can significantly impair dynamic balance control during locomotion [71, 72]. Elderly individuals exhibit a characteristic widening of stride width compared to younger adults [73]. In contrast, PD patients demonstrate pathologically reduced stride width, which is clinically associated with increased fall risk [74]. In our study, striatal inactivation in mice lead to significant reduction in forelimb stride width during FW locomotion, suggesting that the striatum’s critical role in increasing stride width to ensure the balance stability during locomotion.

Rhythmicity reduction is a hallmark motor dysfunction in both PD and HD [75, 76]. The reduced rhythmic locomotion improvement during FW training after inhibiting motor cortical or dopaminergic inputs at the striatum indicate that deficits in motor cortical inputs, and dopaminergic inputs (including the direct and indirect pathway) may all contribute to aberrant locomotion. It is worth mentioning that inhibiting the motor corticostriatal pathway alone decreases drag but not sweep, while inhibiting dopamine receptors (D1 and D2) at the striatum decrease sweep and increase drag. Thus, although motor cortical and dopaminergic inputs to the striatum are important for rhythmic locomotion, they may exert different effects on gait: motor cortical inputs are important for reducing sweep (Fig. 3H), and dopaminergic inputs are critical for reducing drag (Fig. 4G-H). Therapeutic interventions targeting each of these inputs may help promote the recovery of different aspects of rhythmic locomotion. In addition, PD patients with FOG symptoms often exhibit a drag pattern similar to that observed in our study, along with decreased stride length [77, 78]. Given that dopaminergic inputs, not motor cortical inputs, to the striatum are critical for the decrease of drag and the increase of stride length, focusing on the manipulation of dopaminergic inputs including both the direct and indirect pathway may rescue FOG symptom of PD. Furthermore, early PD patients are characterized by stride width decrease, which seems to be correlated to the postural instability and fall susceptibility [76, 79]. Focusing the direct, but not indirect pathway, may rescue the stride width decrease symptom in PD.

In summary, our work highlights multifactorial control of locomotion by the motor corticostriatal pathway and dopaminergic inputs. These findings show that motor cortical and dopaminergic inputs in the striatum coordinately modulate and refine different aspects of locomotion to achieve rhythmic and efficient gaits during motor training. They also suggest that therapeutic approaches targeting different inputs to the striatum are needed to improve gait quality in movement disorders.

## Materials and methods

### Animals

C57BL/6J mice used in this study were purchased from Zhejiang Vital River Laboratory Animal Technology Co. Mice were housed in the animal facility with a maximum of 5 mice per cage at Shanghai Lingang Laboratory. Animals were maintained at 22 ± 2 °C with 12 h light: dark cycle (lights on at 7 am, light off at 7 pm). Food and water were available ad libitum. All mice used in this study were C57BL/6J male mice and randomly assigned to experimental groups.

### Treadmill training

Treadmill training was performed according to previous studies [47, 49, 80–82]. Briefly, before training, head-fixed mice were positioned on a custom-made head-holding plate, with its forelimbs placed on the moving belt and its hindlimbs and hips resting on a custom-made horizontal metal plate to ensure only forelimb locomotion on the treadmill. At the onset of the running session, the belt speed gradually increased from 0 cm/s to 8 cm/s over 3 s. Subsequently, the mice underwent either FW running or BW running training at a constant speed of 8 cm/s for 30 min.

### Gait analysis

To assess forelimb gait variation on the treadmill, the forelimbs of the mice were coated with black ink, and a 42.5 cm-long white paper strip was attached to the treadmill belt, with the starting edge positioned 5 cm ahead of the forelimbs. The treadmill was activated at a fixed speed (8 cm/s) for 5 s during each FW or BW running test trial. Each mouse was tested for 5–8 trials. Similar to previous studies [47, 49, 80], we classified forelimb gait into four categories: Drag (without clear foot lifting and stepping); Sweep (a sweeping pattern without clear paw prints); Wobble (unsteady and irregular steps); Steady run (rhythmic stepping of both right and left forelimbs with clear paw prints) (Supplementary Fig. 4).

Footprints were analyzed offline manually by the following parameters: (1) the fraction of time spent in drag, sweep, wobble or steady run for each mouse over 5–8 trials; (2) the stride length between two left forelimbs with clear pawprints; (3) the stride width between the left and right forelimbs independent of gait (Fig. 1D). Data from 3 to 4 trials were averaged to determine the stride length and width of each mouse.

### In vivo drug administration

6-7-week-old C57BL/6J male mice were anesthetized via an intraperitoneal injection of pentobarbital sodium (80 mg/kg) before drug administration. A custom-made head holder was mounted on top of the mouse skull with cyanoacrylate-based glue and dental acrylic cement, as described in previous studies [47, 49, 80–82]. A high-speed micro-drill were used to thin a circular area (500 mm) of the skull (0.7 mm anterior to bregma, 1.7 mm lateral from midline). A small opening on the skull was made with the tip of a syringe needle to allow the insertion of the glass microelectrode into the brain. Drug injection into the striatum and motor cortex was performed through the electrode with a stereotaxic instrument (RWD life science) and a Nanoliter Microinjection Pump (R-480, RWD life science).

Muscimol hydrobromide (5 mg, Sigma-Aldrich, G019) was dissolved in 50 ml sterile artificial cerebrospinal fluid (ACSF) to prepare a 1 µg/µL working solution. An equal volume of ACSF without muscimol was used as a control. 250 nL of the muscimol solution or vehicle was injected into the striatum (2.6–2.7 mm in depth below the dura) or motor cortex (0.4–0.5 mm in depth below the dura) bilaterally with the injection speed of 0.86 nL/sec.

In the experiment to examine the effect of muscimol on neuronal activity in striatum with fiber photometry, a small incision was first drilled at a site 4 mm horizontally lateral to the implanted optical fiber. 250 nL volume of muscimol was then slowly injected (0.86 nL/sec) into the striatum at a 30-degree angle, with the needle advanced to a depth of 4 mm.

The D1 receptor antagonist SCH23390 hydrochloride (50 ng; MCE, HY-19545 A) was dissolved in 0.2 µL ACSF. The D2 receptor antagonist (s)-(-)-sulpiride (100 mg; MCE, HY-B1059) was dissolved in 2 ml DMSO to make 50 mg/ml stock solution, which was then diluted in ACSF to make the final concentration of 0.05 mg/ml. An equal volume of vehicle without antagonists was used as the control. Using a glass microelectrode, 250 nL of the antagonists or vehicle was injected into the striatum (2.6–2.7 mm in depth below the dura) bilaterally with the injection speed of 0.86 nL/sec.

### Rhodamine B application and spread area analysis

To determine the spread of muscimol, SCH-23,390 or (s)-(-)-sulpiride in the striatum or the spread of muscimol in the motor cortex, 250 nL Dextran rhodamine B (2 µg/µL, dissolved in saline, Invitrogen, D1841) was injected into the striatum or motor cortex of C57BL/6J male mice bilaterally following the same coordinates and injection parameters that were used for drug injection. 30 min after the infusion, mice were sacrificed and the brains were cut into sections at 100 μm with a Vibratome (VT1200S, Leica). Sections were then imaged by using a SLIDEVIEW VS200 research slide scanner (Olympus). The extent of rhodamine B spread was estimated by the line at which the fluorescence was at 20% of its maximum intensity. The spread area was analyzed using the OLYMPUS OlyVIA software. Multiple brain sections were used for quantifying the extent of the rhodamine B spread relative to the area of the striatum or motor cortex: Area _(rhdamine B)_/Area _(striatum)_ or Area _(rhdamine B)_/Area _(motor cortex)_.

### Virus injection

To label axonal projections from the motor cortex to the striatum, 150 nL of 10-fold diluted AAV2/9-hSyn-EGFP-3xFLAG-WPRE virus (1.58E + 13 vg/mL, OBIO, Shanghai, China) was slowly injected bilaterally into the motor cortex of 3-week-old C57BL/6J male mice. For optogenetics experiments to inactivate the motor corticostriatal pathway, 150 nL concentrated AAV2/9-hsyn-eNpHR3.0-EYFP-WPRE-hGH-pA virus (5.61E + 12 vg/mL, BrainVTA, Wuhan, China) was slowly injected bilaterally into the primary motor cortex of 3-week-old C57BL/6J male mice (bregma: 1.0 mm; lateral: 1.5 mm; ventral: 0.5 mm). The injected volume (150 nL) of AAV for halorhodopsin expression was smaller than that of muscimol (250 nL). As the result, halorhodopsin expression was predominantly restricted to the primary motor cortex.

For imaging neuronal activity of the motor cortex, 200 nL of 10-fold diluted AAV9-CaMkIIα-GCaMP6s virus (3.71 E + 13 vg/mL, WZ Bioscience Inc, Jinan, China) was injected into the motor cortex unilaterally (bregma: 1.0 mm; lateral: 1.5 mm; ventral: 0.5 mm). For measuring neuronal activity in the striatum, 300 nL of 10-fold diluted AAV2/9-syn-jGCaMP8m-WPRE virus (1.30E + 14 vg/mL, Lingang Laboratory, Shanghai, China) were slowly injected into the striatum unilaterally in 5-week-old C57BL/6J male mice (bregma: 0.7 mm; lateral: 2 mm; ventral: 2.8–2.9 mm). The injection speed was set at 0.16 nL/sec using a Nanoliter Microinjection Pump (R-480, RWD life science). The injection electrode was withdrawn 5 min after the end of the injection. After the injection, the incision was closed with suture and the animals were returned to the cage before next experiments.

### In vivo optogenetic manipulation

2 weeks after virus injection, AAV-eNpHR3.0, or AAV-EGFP injected mice received bilateral implantation of optical fibers (O.D. = 200 μm, Φ = 1.25 mm, NA = 0.37, 3 mm long; Hangzhou Sanshi Biotechnology CO., LTD, China.) in the DLS with 5 degree angle (bregma: 0.6 mm; lateral: 2.75 mm; ventral: 2.55 mm). Optical fiber was fixed to the skull with dental cement, then a custom-made head holder (two parallel micro metal bars) was mounted on top of the skull with cyanoacrylate-based glue and dental acrylic cement as described previously [47, 81]. Mice were kept on a heating pad until fully recovered from anesthesia. Mice were allowed to recover for 7 days after surgery.

During the behavioral tests/training, 589 nm light was used to illuminate the projections from the motor cortex to DLS (eNpHR3.0/EGFP: wave width: 20 ms; frequency:50 Hz; power: 10 mW; duration 100 ms, delay time 100 ms, run time 1800 s). The location of optical fiber in the striatum was confirmed in all animals after preparing coronal Sect. (100 μm) with the implantation sites using a Vibratome (VT1200S, Leica) and imaging with a SLIDEVIEW VS200 research slide scanner (Olympus).

### In vivo fiber photometry recording

Immediately following AAV-syn-jGCaMP8m virus injection, mice received unilateral implantation of an optical fiber (O.D. = 200 μm, Φ = 1.25 mm, NA = 0.37, 3 mm long; Hangzhou Sanshi Biotechnology CO., LTD, China.) in the striatum (bregma: 0.5 mm; lateral: 2 mm; ventral: 2.6–2.7 mm). The optical fiber was securely attached to the skull using dental cement, and a custom-made head holder (two parallel micro metal bars) was mounted on top of the skull [47, 81].

2–3 weeks after virus expression, three color single channel optical fiber photometry system (405/470/580 nm, Thinker Tech Nanjing Biotech Inc, Nanjing, China) was used for recording GCaMP8m fluorescence signals. The light beam from a 405 and 470-nm laser (OBIS 488LS; Coherent, Santa Clara, CA, USA) was reflected with a dichroic mirror (MD498; Thorlabs) and focused with a 10-x objective lens (NA = 0.3; Olympus, Tokyo, Japan), then coupled to an optical commutator (Doric Lenses). An optical fiber (O.D. = 200 μm O.D., NA = 0.37, 3 mm long; Hangzhou Sanshi Biotechnology CO., LTD, China.) guided the light between the commutator and the implanted optical fiber. To minimize GCaMP8m bleaching, the laser power at the tip of the optical fiber was adjusted to a low level (0.04–0.05 mW). The GCaMP8m fluorescence emission was bandpass filtered (MF525-39, Thorlabs) and detected by a photomultiplier tube (R3896, Hamamatsu). An amplifier (C7319, Hamamatsu) was used to convert the photomultiplier tube current output to voltage, which was further filtered through a low-pass filter (35 Hz cut-off; Brownlee 440). The analog voltage signals were digitized at 500 Hz and recorded by fiber photometry software (TrippleCoLo rMultichannel_TP_OG_20230202_3D). Neuronal activity of the striatum was recorded during the period of 30 s quite awake (resting) and 100 s FW running state before and 30 min after muscimol injection in the striatum using the same experimental protocol.

Fiber-photometric recording data were exported as MATLAB files for further analysis. All the raw fluorescence data (F) were smoothed with a moving average filter (10 ms span) and then segmented and aligned to the onset of behavioral events within individual trials or bouts. The fluorescence change values (ΔF/F) were calculated as (F–F0)/(F_0_–V_offset_), where F_0_ is the baseline fluorescence signal averaged over a 5 s-time window prior to FW running and V_offset_ is the fluorescence signal recorded before the cannula was connected to the optical fiber.

### Ca^2+^ imaging of pyramidal neurons in the motor cortex

Prior to in vivo two-photon Ca^2+^ imaging of pyramidal neurons (PNs), the forelimb region of the motor cortex to be imaged was marked based on stereotactic coordinates (1.3 mm anterior to bregma, 1.2 mm lateral from midline) in head-restrained mice [83]. A high-speed micro-drill was used to thin a circular area (300 mm) of the skull to a thickness of less than 20 μm. Ca^2+^ imaging was performed through a thinned-skull window as described previously [47, 49, 81]. Before Ca^2+^ imaging, mice were habituated for at least 5 min under the microscope to reduce potential stress. The InSight X3 laser (Spectra Physics) was tuned to 920 nm, and time-lapse imaging of PNs were obtained using an Olympus two-photon microscope (FVMPE-RS) with a 1.05 N.A. 25X objective immersed in ACSF. The average laser power on the sample was 20–30 mW for imaging. All the time-lapse Ca^2+^ images were acquired at a frame rate of 2 Hz (2-mspixel dwell time).

Ca^2+^ imaging was performed during the period of 30 s quiet awake (resting) and 120 s FW running state. 30 min after muscimol injection in the motor cortex, Ca^2+^ activity of the same population of PNs were recorded again. Image acquisition was performed using FV31S-SW software and analyzed post hoc using NIH ImageJ and MATLAB (Mathworks) software.

### Ca^2+^ image analysis

Before data analysis, all time-lapse images from each individual field of view were motion corrected to minimize the impact of horizontal movement caused by running, and facilitate subsequent imaging processing [84]. During running trials, the lateral movement of the images was typically less than 1 μm. Image correction was done using codes in MATLAB (https://github.com/wujunjun/calcium-trace-parameter-calculation).

Visually identifiable and clear neurons were manually outlined as regions of interests (ROIs), similar to previous studies [47, 49]. The fluorescence intensity (F) of each ROI was calculated as the average fluorescence intensity of all pixels within the ROI, with the background fluorescence value from blood vessels subtracted. This yields the fluorescence curve of each ROI over time.

The baseline (F_0_) of the fluorescence curve is determined by averaging the lowest 8% of fluorescence intensity values across the total frames of the curve. The change in fluorescence intensity (ΔF/F_0_) is calculated as: ΔF/F_0_ = (F-F_0_)/F_0_ × 100%.

The threshold is set at three times the standard deviation of the baseline fluorescence (3*SD). Signals exceeding this threshold are considered Ca^2+^ events and recorded as PNs Ca^2+^ activity. The total Ca^2+^ activity of a neuron is defined as the sum of all threshold-exceeding Ca^2+^ signals within the imaging period. PNs Ca^2+^ activity analysis was done using custom codes in MATLAB (https://github.com/wujunjun/calcium-trace-parameter-calculation).

### Quantification and statistical analysis

All data in this study underwent normality testing (Single-sample Kolmogorov-Smirnov test) to assess whether the data followed a normal distribution. Parametric tests were used for normally distributed data, while non-parametric tests were applied to non-normally distributed data. Since the behavioral experiments and neuronal Ca^2+^ activity data in this study did not conform to a normal distribution, paired comparisons of the same sample at different time points were analyzed using non-parametric tests: Wilcoxon matched-pairs signed rank test. Comparisons between two independent samples were conducted using the Mann-Whitney U test. All experimental data are presented as mean ± SEM. *P* > 0.05 was considered statistically non-significant (n.s.), while *P* < 0.05 were deemed statistically significant (*P < 0.05, **P < 0.01, ***P < 0.001, ****P < 0.0001). The software used for data analysis included ImageJ (National Institutes of Health, Bethesda, MD), MATLAB (2023 version), and GraphPad Prism 10.1.2. Figures were prepared using Adobe Illustrator and Photoshop (2023 version).

## Electronic Supplementary Material

Below is the link to the electronic supplementary material.

Supplementary Material 1

## Data Availability

No datasets were generated or analysed during the current study.

## Abbreviations

MLR: Mesencephalic locomotor region
CPGs: Central pattern generators
PD: Parkinson’s disease
HD: Huntington’s disease
MSNs: Medium spiny neurons
FOG: Freezing of gait
dSPNs: Direct pathway striatal projection neurons
iSPNs: Indirect pathway projection neurons
FW: Forward
BW: Backward
SNr: Substantia nigra pars reticulata
GPi: Globus pallidus internus
DLS: Dorsal lateral striatum
SNc: Substantia nigra pars compacta
VTA: Ventral tegmental area
ACSF: Artificial cerebrospinal fluid
PNs: Pyramidal neurons
ROIs: Regions of interests
MC: Motor cortex
