# Supplementary material for: The regulation of rhythmic locomotion by motor cortical and dopaminergic inputs in the mouse striatum
Source: Mol Brain. 2025 Jul 16;18:63. doi: 10.1186/s13041-025-01232-8 (PMC12269160; doi:10.1186/s13041-025-01232-8)
Supplement: Supplementary file 1 — Supplementary Material 1 [file 13041_2025_1232_MOESM1_ESM.docx]

**Supplementary figures**

**
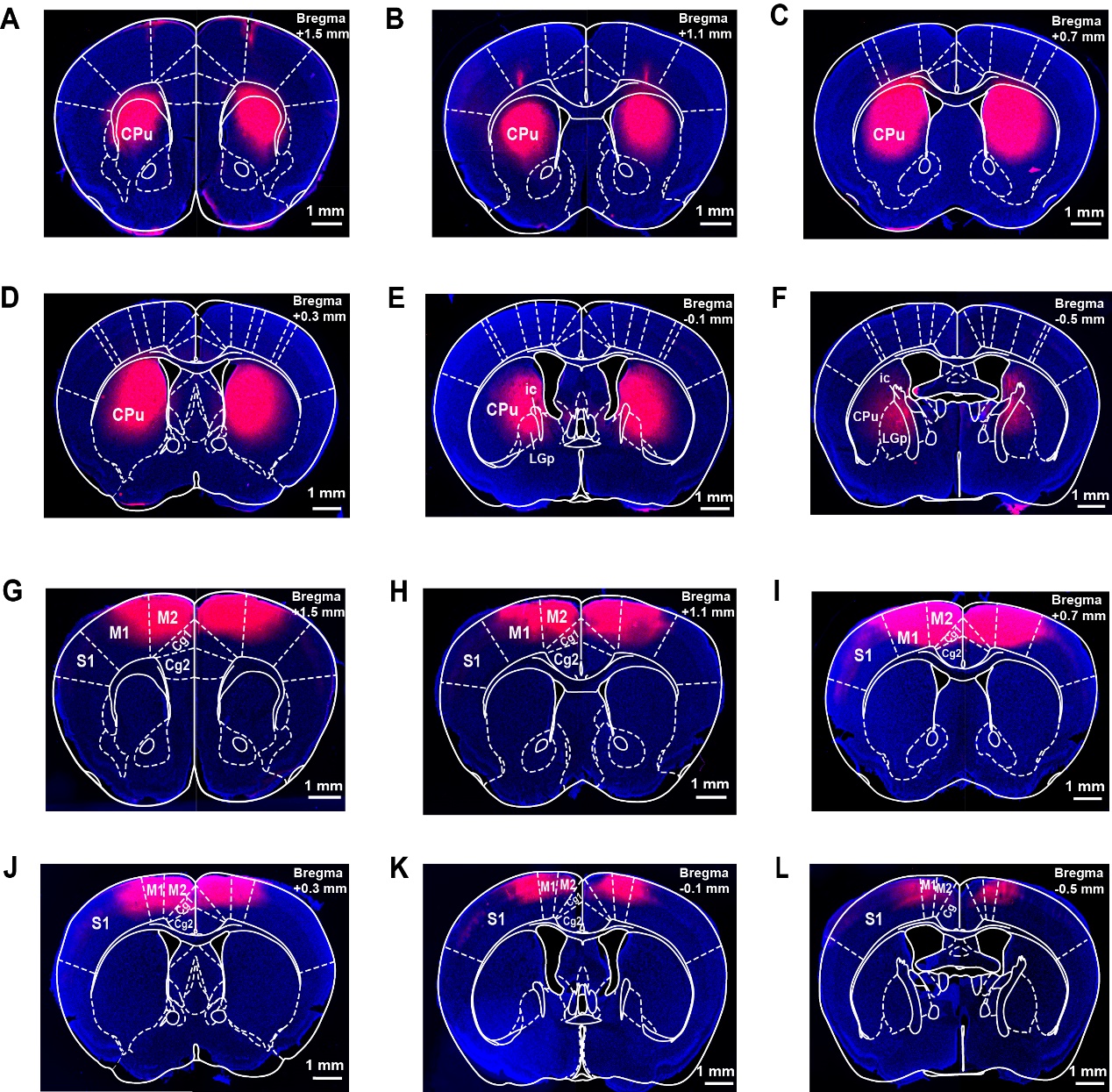
**

**Fig S1. The extent of rhodamine B spread in the striatum and motor cortex, 30 minutes after injection.**

(A-L) Representative brain sections showing the diffusion of rhodamine B 30 minutes after injection.

Rhodamine B spread was analyzed in multiple sections covering the striatum (A-F) . On average, 64.28 ± 6.29% of striatum was covered by Rhodamine B (n = 4 mice).

Rhodamine B spread was analyzed in multiple sections covering the region of the motor cortex (from Bregma -0.5 to 1.5 mm) that regulates forelimb movement (G-L). On average, 71.62 ± 6.64% of motor cortical region controlling forelimb movement was covered by Rhodamine B. In addition, 11.94 ± 1.43 % of the primary somatosensory cortex (S1) and 27.07 ± 4.95 % of the cingulate area were covered by Rhodamine B, 30 minutes after the injection (n = 3 mice). Scale bar, 1 mm.

**
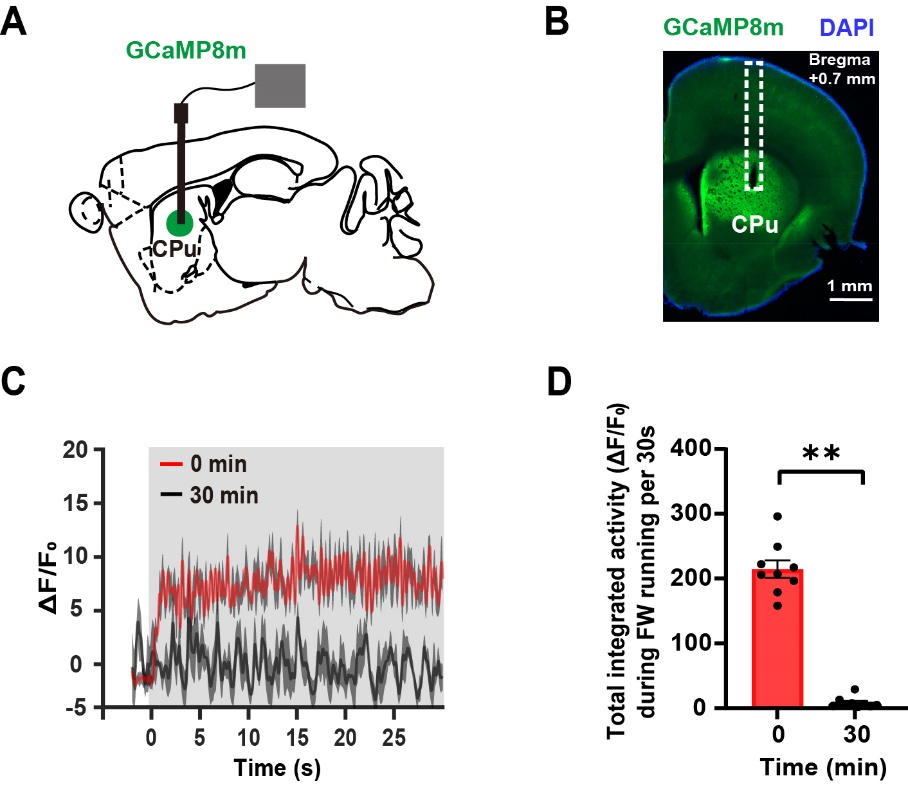
**

**Fig S2. Neuronal activity in the striatum was significantly reduced 30 minutes after muscimol injection.**

1. Experimental setup: GCaMP8m virus was injected into the striatum and a photometry fiber was implanted.
2. Representative image showing GCaMP8m expression and optic fiber implant. GCaMP8m expression is in green and DAPI is in blue. Scale bar, 1 mm.
3. Examples of neuronal activity during FW running before (red line) and 30 minutes after muscimol injection (black line) in the striatum. Gray lines: single trials; red/black line: average.
4. The level of neuronal activity (ΔF/F_0_) in the striatum during FW running significantly decreased 30 minutes after muscimol injection (n = 9 trials from 3 mice). All data are presented as mean ± S.E.M. **P < 0.01. Wilcoxon test. See methods for statistical details.


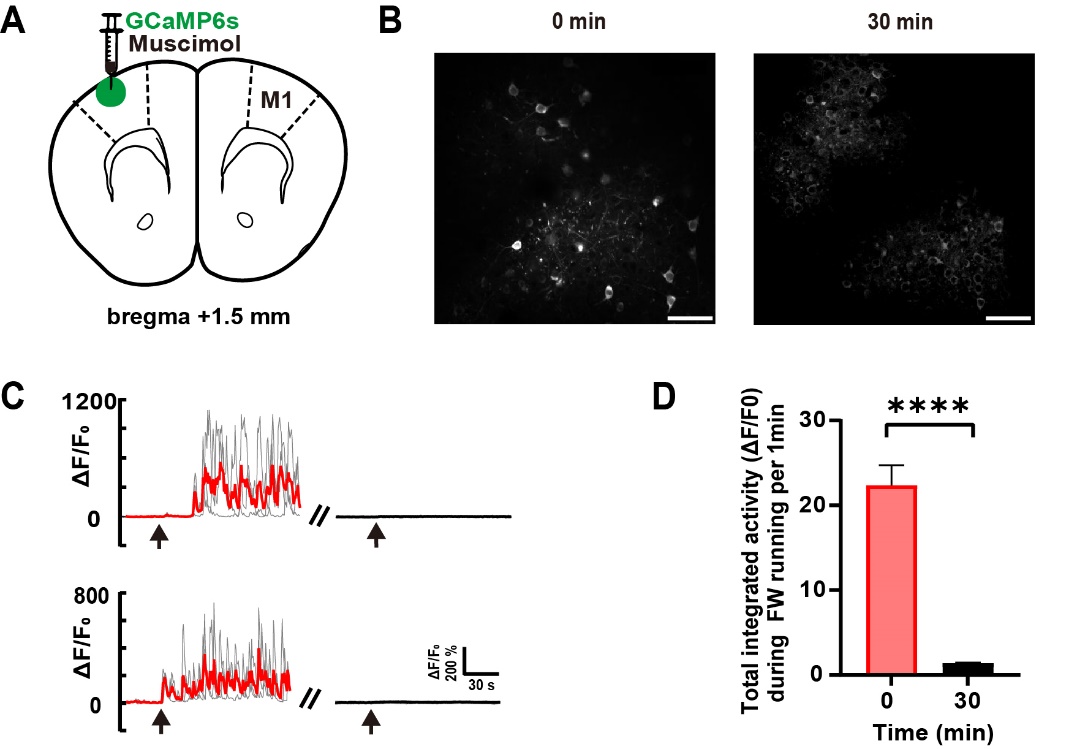


**Fig S3. Reduced neuronal activity in the motor cortex 30 minutes after muscimol injection.**

1. Schematic showing the injection of AAV virus expressing GCaMP6s and muscimol into the motor cortex.
2. Two photon images of pyramidal neuron (PN) Ca^2+^ activity in response to FW running before (left) and 30 minutes after muscimol injection (right) in the motor cortex. Scale bar, 60 μm.
3. Two examples of PN Ca^2+^ activity in response to FW running (arrow: treadmill on) before (left) and 30 minutes after muscimol injection (right) in the motor cortex. Gray lines: single trials; red/black line: average.
4. The level of PN Ca^2+^ activity (ΔF/F_0_) in the motor cortex during FW running significantly decreased 30 minutes after muscimol injection (n = 164 cells from 4 mice). All data are presented as mean ± S.E.M. ****P < 0.0001. Wilcoxon test. See methods for statistical details.


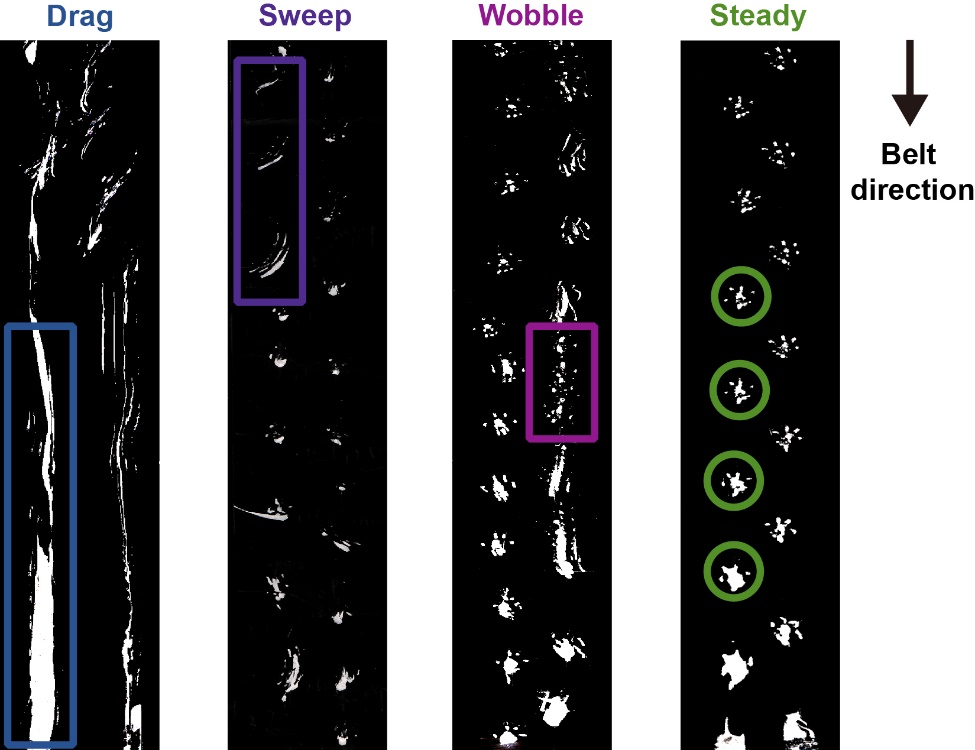


**Fig S4. Forelimb gait classification during forward running locomotion.**

Representative forelimb gait during forward running on the treadmill. Four types of forelimb gait (drag, sweep, wobble and steady run) were observed.
